# Effects of the healing activity of rosemary-of-Chapada (Lippia gracilis Schauer) on cutaneous lesions in rats

**DOI:** 10.1590/acb370104

**Published:** 2022-04-08

**Authors:** Apolônia Agnes Vilar de Carvalho Bulhões, Lígia Reis de Moura Estevão, Rinaldo Florencio-Silva, Ricardo Santos Simoes, Ana Greice Borba Leite, Dayana Maria Serafim da Silva Cunha, Clécio Souza Ramos, Érica Bruna de Andrade Soares, Marcela Barbosa D’Emery, Cláudio Augusto Gomes da Câmara, Joaquim Evêncio-Neto

**Affiliations:** 1 Doctoral Fellow. Universidade Federal Rural de Pernambuco – Postgraduate Program in Veterinary Medicine – Department of Animal Morphology and Physiology – Laboratory of Histology – Recife (PE), Brazil.; 2 Postdoctoral Fellow. Universidade Federal Rural de Pernambuco – Postgraduate Program in Animal Bioscience – Department of Animal Morphology and Physiology – Laboratory of Histology – Recife (PE), Brazil.; 3 Postdoctoral Fellow. Universidade Federal de São Paulo – Department of Morphology and Genetics – São Paulo (SP), Brazil.; 4 Full Professor. Centro Universitário FACOL – Department of Veterinary Medicine – Vitória de Santo Antão (PE), Brazil.; 5 MSc. Universidade Federal Rural de Pernambuco – Department of Animal Morphology and Physiology – Laboratory of Histology – Recife (PE), Brazil.; 6 PhD. Universidade Federal Rural de Pernambuco – Department of Chemistry – Laboratory of Bioactive Natural Products –Recife (PE), Brazil.; 7 Full Professor. Universidade Federal Rural de Pernambuco – Department of Animal Morphology and Physiology – Laboratory of Histology – Recife (PE), Brazil.

**Keywords:** Oils, Volatile, Wound Healing, Phytotherapy, Rats

## Abstract

**Purpose:**

To evaluate the effects of rosemary leaf essential oil-based ointments on the healing of rat skin lesions.

**Methods:**

Sixty adult male rats, with dorsal excisional skin wounds made surgically under anesthesia, were divided into three groups (n = 20): Sham group (untreated wounds); control group (CG, wounds treated with vehicle); and essential oil (EO) treated group (wounds treated with essential oil-based ointments), administered topically once daily. Skin wounds were evaluated at 4, 7, 14, and 21 days after EO or vehicle treatments. Lesions were analyzed macroscopically for the contraction degree. Formalin-fixed paraffin-embedded sections of skin wounds were used for histopathological evaluation.

**Results:**

Macroscopic evaluation showed wounds edges with thin crust without firmness and yellowish color, along with an improvement in wound contraction in EO group when compared to the other groups. A reduced inflammatory reaction, along with newly formed small diameter capillaries and more organized and elongated collagen fibers, were more frequently observed in EO group than in the other groups. Moreover, blood vessel number and collagen fibers density were significantly higher in EO group.

**Conclusions:**

Skin lesion treatment with rosemary leaf essential oil-based ointments accelerates the initial stages of healing, reduces inflammation, and increases angiogenesis, collagen fibers density, and wound contraction in rats.

## Introduction

Wound healing is a complex process that involves cell organization, chemical signals and extracellular matrix remodeling, with the aim of restoring tissue structure and function. Wound treatment aims to quickly close the lesion in order to obtain a scar, maintaining tissue function and satisfactory esthetics^1^. For this purpose, the use of natural substances extracted from plants, many of which are native to Brazil, have been shown to be effective in improving skin wound healing^2^^-^ 7. Among these plants, the hydroalcoholic extract of *Sphagneticola trilobata* 2, the oil of mastic tree (*Schinus terebinthifolius* Raddi)^3^^-^ 5 and the hydroalcoholic extract of wild plum (*Ximenia americana*)^6^ stand out, along with a wide variety of medicinal plants described elsewhere^7^.

In Northeastern Brazil, the species *Lippia gracilis* Schauer (*L. gracilis*) is used by traditional communities as a natural resource for the treatment of respiratory problems, such as sinusitis, bronchitis, nasal congestion, and pain^8^. Moreover, its essential oil has been topically used for treatment of skin diseases, burns, wounds, and ulcers^9^. Popularly known as rosemary-of-Chapada, *L. gracilis* is a species of the Verbenaceae family native to the *caatinga* biome, located in Northeastern Brazil^10^. It is rich in essential oil predominantly composed of thymol and carvacrol^11^, which have been shown antimicrobial^11^^-^ 15, cytotoxic^16^, anti-inflammatory, and antinociceptive^17^^-^ 18 activities. However, the effects of *L. gracilis* leaf-based essential oil on the healing of skin lesions are poorly understood.

Assuming that there is great economic and scientific interest in the discovery of new substances as alternative therapies for the treatment of skin lesions, combined with the biological activities presented by the essential oil of *L. gracilis*, it is important to evaluate its healing effect. Thus, the aim of this study was to evaluate the effects of ointment consisting of 10% rosemary-of-Chapada essential oil on the healing of skin lesions in rats.

## Methods

### Animals and acclimatization

This study was approved by the Ethics Committee on the Use of Animals (CEUA) of the Rural University of Pernambuco (UFRPE) at protocol no 57/2017.

The experiment was carried out at the Department of Animal Morphology and Physiology (DMFA) of the UFRPE. Sixty male albino Wistar rats (*Rattus norvergicus albinus*), with 3 months of age and body weight between 250 and 300 g, were purchased from the vivarium of the DMFA of UFRPE. The animals were kept in individual cages at a room with controlled temperature (23 ± 2 °C) and humidity (50 ± 10%), under a 12/12 h light/dark cycle (lightning period beginning at 07 a.m.). The rats received Presence chow and water *ad libitum*. All animal care and experimental procedures were conducted in accordance with the *Guidelines for Animal Experimentation of the National Council for Control of Animal Experimentation* 19.

### Botanical material and preparation of formulations

Rosemary-of-Chapada leaves (*L. gracilis* Schauer) were collected in January 2017, in the morning, at the Campus Dois Irmãos of UFRPE, latitude (S) 8° 1´ 0.52˝ and longitude (W) 34° 95´ 0.1˝. A specimen of the botanical material was stored at the Professor Vasconcelos Sobrinho Herbarium at UFRPE, registration number: 53.610. The process of obtaining the essential oil was carried out at the Laboratory of Bioactive Natural Products of the Department of Chemistry at UFRPE, where the fresh leaves were weighed (1000 g), washed, crushed, and submitted to the hydrodistillation technique using the Clevenger-type device during 2 h. Afterwards, the total amount of oil was calculated based on the weight of fresh leaves and the result was expressed as a percentage. The essential oil was stored in a hermetically closed glass container and kept under refrigeration (4 °C) until chemical analysis and later used in the experiment.

The chemical analysis of the oil was performed at the Research Support Center (Cenapesq) of UFRPE, by gas chromatography coupled with mass spectrometry (CG/MS). The identification was made based on the comparison of retention indices^20^^,^ 21, as well as by computer comparison of the obtained mass spectrum with those contained in the National Institute of Standards and Technology (NIST) mass spectra library of the CG-EM21 dataset. Two formulations were prepared at the Pharmacology Laboratory at UFRPE. The control formulation consisted only of the vehicle solution (70% petroleum jelly and 30% anhydrous lanolin). The test formulation consisted of essential oil from the leaves of *L. gracilis* Schauer at 10% diluted into the vehicle solution.

### Surgical procedure

The animals received dissociative anesthesia of 2% xylazine hydrochloride (10 mg·kg^–1^) and 10% ketamine hydrochloride (60 mg·kg^–1^), administered intramuscularly. Subsequently, trichotomy was performed in the right and left dorsolateral thoracic regions, and antisepsis was performed with topical 2% chlorhexidine. Two excisional wounds were made on the back of each rat with the aid of a disposable 8-mm diameter dermatological punch, exposing the adjacent muscle fascia. After surgical procedure, the animals were divided into three groups (n = 20): Sham group: did not receive topical treatment; control group (CG): received daily topical application of the ointment consisting only of the vehicle (70% petroleum jelly and 30% anhydrous lanolin); and the essential oil treated group (EO): received daily topical application of the ointment consisting of 10% essential oil from the leaves of *L. gracilis*, diluted in the vehicle solution. Treatment was performed by applying 0.2 mL of ointment or vehicle solution, topically, on each wound with a disposable syringe to ensure that the amount used was the same for all animals. Then, each group were further divided into four subgroups (G4, G7, G14 and G21, n = 5 each), which represent the time points where skin wounds were evaluated after topical application of essential oil or vehicle solution (4, 7, 14, and 21 days after operation, respectively).

### Clinical evaluation of animals and wound closure assessment

Clinical evaluations were performed immediately after surgical procedure and continued daily. The general condition of the animals and the presence of hyperemia, edema, pain, secretion, pruritus, crust, contraction, granulation tissue, and scar tissue at the wound site were observed. The wound area was measured throughout the experiment on days 0, 4, 7, 14, and 21 postoperative days with the aid of a digital millimeter caliper (King Tools). Results were expressed as a percentage of closure relative to the original wound size, as shown in Eq. 1:


$$wound\;cibtractuib=original\;wound\;area-\frac{present\;wound\;area}{total\;wound\;area}\times 100$$ (1)

### Histomorphological and histomorphometric evaluations

After macroscopic evaluation of wounds on days 4, 7, 14, and 21 PO, skin fragments were collected and fixed for 24 h in 10% formalin buffered with a 0.1 mol L^–1^ sodium phosphate solution at pH 7.2. Then, the specimens were dehydrated in ascending concentrations of ethanol, cleared in xylene, and embedded in paraffin. Sections (5 μm thick) from paraffin-embedded wound blocks were stained with hematoxylin/eosin (H/E) or Gomori’s trichrome, and used for histomorphological and histomorphometric analyses. Images of the stained sections were digitized using a trinocular light microscope (Leica DM500) attached to a HD camera (Leica ICC50) and a software-equipped computer (Leica LAS EZ software).

Images of five fields per wound were obtained at 100× or at 400× magnifications for histomorphological and histomorphometric evaluations, respectively. For histomorphological evaluation, the following parameters were evaluated at all time points (4, 7, 14, and 21): crust formation, inflammatory infiltrate, extracellular matrix deposition (ECM), vascular formation, and reepithelialization^21^. The analysis consisted of a slide scan and a comparative analysis of the groups, differentiating in scores from 0 to 3 for each parameter evaluated. Zero (0) represented the absence of the tested parameter (whole skin); 1, discreet; 2, moderate; and 3, severe (Table 1)^22^.

**Table 1:** Morphological evaluation of wound healing events in the excisional wound healing model in rats^22^.

Morphological scores	Score	Parameter
Inflammation	0	WHOLE SKIN: absence of inflammation
	1	DISCRETE: presence of few inflammatory cells
	2	MODERATE: many inflammatory cells
	3	SEVERE: exaggerated inflammatory cellularity
Scab	0	ABSENCE
	1	DISCRETE
	2	MODERATE
	3	SEVERE
Extracellular matrix deposition	0	WHOLE SKIN: whole extracellular matrix
	1	DISCRETE: incomplete presence of extracellular matrix
	2	MODERATE: presence of extracellular matrix in the whole wound area (identified by many fibroblasts, and thin collagen fibers)
	3	HIGH: presence of extracellular matrix in the whole wound area (identified by few fibroblasts, and thick collagen fibers)
Vascularization	0	WHOLE SKIN: normal vascularization
	1	DISCRETE VASCULAR FORMATION
	2	MODERATE VASCULAR FORMATION
	3	HIGH VASCULAR FORMATION
Epithelialization	0	WHOLE SKIN: whole epithelium
	1	DISCRETE: partial epithelialization with a small new epithelial layer (the epithelial tongue occupies, at most, 1/3 of the wound gap)
	2	MODERATE: partial epithelialization with a longer new epithelial layer (the epithelial tongue occupies more than 1/3 of the wound gap)
	3	COMPLETE epithelialization

For histomorphometry, angiogenesis was determined by the quantification of blood vessels profile in H/E-stained sections. Therefore, images of five fields of newly formed granulation tissue were randomly captured moving from left to right, and encompassing the entire extent of the scar area of each slide on a 50-μm scale at all time points analyzed^5^. Images were analyzed using the ImageLab software (version 6.0). To determine the density of collagen fibers, the same image capture methodology was used (five fields/slide, 50 μm scale, total of five slides/group), in Gomori’s trichrome-stained sections. The images obtained were analyzed using the GIMP software (version 2.8).

### Statistical design and analysis

Statistical analysis was carried out using the GraphPad Prism 5.0 software and the results were presented as the mean ± standard error (SEM). Comparisons between the three groups were performed using Student’s t-test for multiple comparisons. Two-way analysis of variance (ANOVA) was used for graphical lines and to verify the interaction between independent variables in time and treatment, followed by Bonferroni post-test. Statistical significance was set at p < 0.05.

## Results

### Chemical profile and yield of essential oil leaves from L. gracilis Schauer

**Figure 1:**
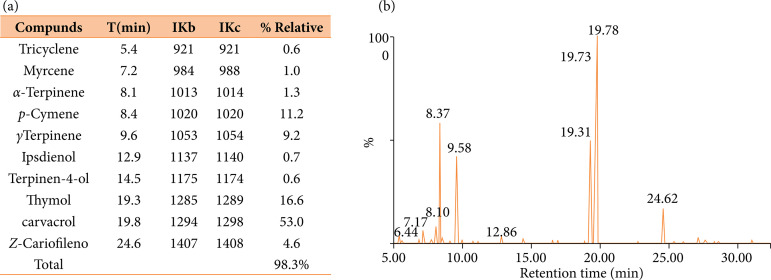
**(a)** Chemical composition of the essential oil obtained from *L. gracilis* Schauer leaves. Compounds are listed in order of election in a nonpolar DB-5 column; retention indices calculated through the retention times in relation to the n-alkanes series (C9-C19) in a Phenomenex ZB5-MS 30 m × 0.25 μ column; % Relative: relative percentage of the compound. **(b)** GC-MS chromatogram of the essential oil obtained from *L. gracilis* Schauer leaves.

The essential oil from *L. gracilis* leaves obtained a yield of 1.68%. Ten compounds were identified, which represent 98.38% of the essential oil; their retention indices and percentages are shown in Fig. 1a. The components identified with the highest percentage of concentration according to their retention indices^21^ were: carvacrol (53%), thymol (16.1%), p-cymene (11.2 %), and γ-terpinene (9.2%). Additionally, a representative chromatogram of the essential oil from the leaves of *L. gracilis* is shown in Fig. 1b.

### Contraction of wounds

**Figure 2:**
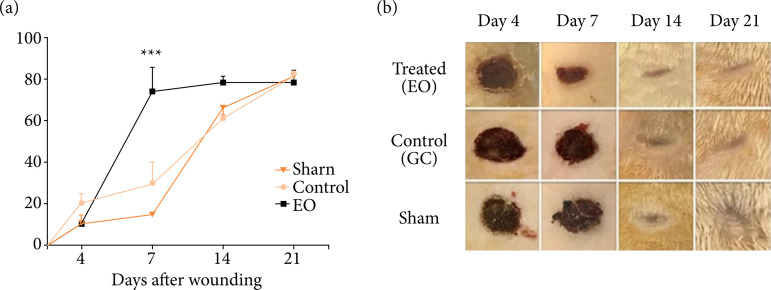
Wound contraction was improved in the group treated with essential oil from *L. gracilis* leaves (EO). **(a)** Wound closure kinetics in EO, CG and Sham groups. **(b)** Representative macroscopic images of the wounds from each group. Wound contraction results were expressed as percentage of closure in relation to the original size (1 – [wound area] / [original wound area] × 100). Data are represented as mean ± SEM; n = 5 rats in each time point. *** p < 0.001, on the 7th postoperative day (Two-way ANOVA).

Wound contraction occurred in all groups from day four after operation. However, the EO group showed a significant increase in the percentage of wound contraction from day 7 after operation, when compared to the CG and Sham groups. Approximately 72.5% (EO), 30% (CG), and 12.5% (Sham); p < 0.001 (Fig. 2a).

### Macroscopic wound evaluation

Macroscopically, the wounds showed few or no hyperemia and edema as well as no bleeding in all animals during the experiment. All groups showed a fibrinoleukocyte layer. However, that layer was more evident in the Sham group from the 4th postoperative day onwards, evolving to thick, dark, firm and irregular crust that covered the entire wound area, which was similar to the CG group. Meanwhile, the EO group presented a thin layer crust, without firmness and a yellowish color on the wound edges. All wounds from the 14th postoperative day onwards showed no crust, and, on the 21st postoperative day, they were completely reepithelialized (Fig. 2b).

### Histomorphological and histomorphometric evaluation of wounds

**Figure 3:**
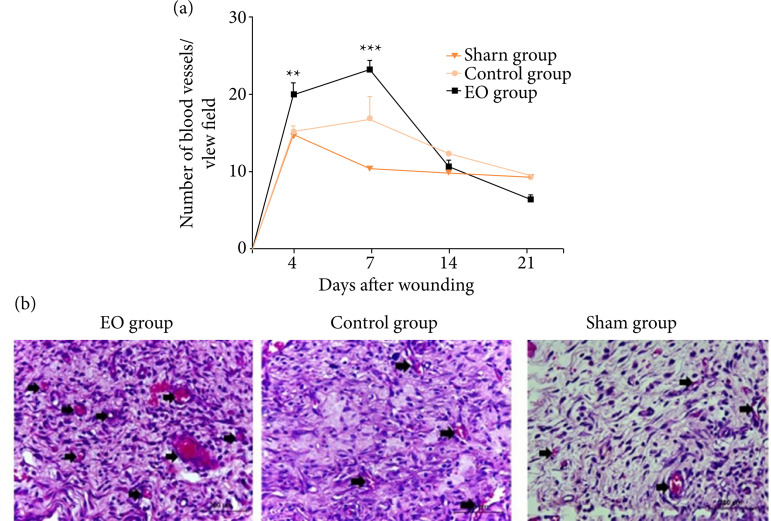
Angiogenesis was increased in the essential oil treated group (EO). **(a)** Kinetics of blood vessel count. **(b)** Representative photomicrographs of H/E-stained histological sections 7 days after wounding. Arrows indicate blood vessels. Data are expressed as mean ± SEM, n = 5 for each time point and group. ** p < 0.01 – EO group versus CG and Sham groups at 4th day after wounding; *** p < 0.001 – EO group versus CG and Sham groups 7 days after wounding. Two-way ANOVA. Scale bar: 200 μm.

**Figure 4:**
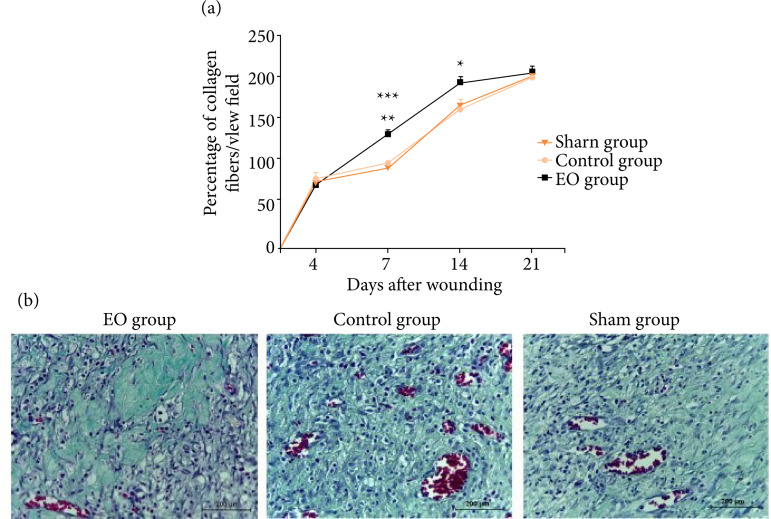
An increased collagen fibers deposition was noticed in wounds of EO group. **(a)** Kinetics of the percentage of collagen fibers. **(b)** Representative photomicrographs of Gomori’s trichrome-stained sections at the 7th postoperative day. Morphologically, collagen fibers presented thicker and more organized in the EO group, as compared to CG and Sham groups. Data are expressed as mean ± SEM, n = 5 for each time point and group. ** p < 0.01 – EO group versus CG; ***p < 0.001 – EO group versus Sham at the 7th postoperative day, and *p < 0.05 – EO group versus CG and Sham at the 14th postoperative day. Two-way ANOVA. Scale bar: 200 μm.

At the 4th postoperative day, crust formation was mild to moderate in the EO group, and moderate to complete in the CG and Sham groups. A discrete epithelialization was noticed in the EO and CG groups, equivalent to the presence of a discrete epithelial tongue. Meanwhile, epithelialization was absent in the Sham group. Moreover, all groups presented a mild collagen differing in the inflammatory process, which was moderate to intense in the EO group and intense in the CG and Sham groups. At the 7th postoperative day, crust was mild, moderate, and intense in the EO, CG and Sham groups, respectively, whereas little reepithelialization with a slight epithelial tongue (score 1) was seen in all wounds. An extracellular matrix filling the entire repair area, with abundant fibroblasts and inflammatory infiltrate of moderate characteristics in the EO, and moderate to intense in the CG and Sham groups was also noticed at the 7th postoperative day. Moreover, the presence of neoformed blood vessels was more prominent in the EO group at the 4th and 7th postoperative days (Figs. 3 and 4, Table 2).

At the 14th postoperative day, all wounds had no crust and exhibited completely reepithelialized areas. A completely formed extracellular matrix with less cellularity and many thick bundles of collagen fibers was mainly observed in the EO group. Meanwhile, an intense extracellular matrix with thinner and more disorganized collagen fibers, along with moderate to intense inflammatory infiltrate, was observed in the CG and Sham groups, respectively. At the 21th postoperative day, the wounds of all groups were healed, epithelialized, without crusts and with little cellularity. The wounds of EO group presented a granulation tissue with thicker collagen fibers oriented along its tension line than CG and Sham groups. A mild inflammatory infiltrate was noticed in the CG and EO groups, while it was mild to moderate in the Sham group (Table 2).

Histomorphometry showed that at the 4th and 7th postoperative days, a higher number of blood vessel profiles was observed in EO group than the CG (p < 0.01) and Sham (p < 0.001) groups (Fig. 3). The Gomori’s trichrome stained sections revealed that at the 7th and 14th postoperative days, the density of collagen fibers was higher in the EO group as compared to CG (p < 0.01) and Sham (p < 0.001) groups, remaining significantly higher at the 14th postoperative day (p < 0.05) (Fig. 4).

**Table 2:** Mean scores of the histomorphological evaluation of skin wounds in rats at days 4, 7, 14, and 21 after operation. Group treated with essential oil from *L. gracilis* leaves (EO), control group treated only with vehicle solution (CG) and Sham group, which received no treatment.

Days postoperation (PO)	Groups	Crust formation	Reepithelializedarea	Inflammatory cells	Extracellular matrix
4th day PO	EO	1.5	0.4	2.4	0.6
	CG	2.6	0.4	3.0	0.4
	Sham	2.8	0	2.9	0.2
7th day PO	EO	1.2	1.0	2.1	2.4
	CG	2.0	1.0	2.6	2.0
	Sham	2.7	1.0	2.5	1.8
14th day PO	EO	0	3	0.8	2.7
	CG	0	3	2.1	2.5
	Sham	0	3	1.9	2.4
21th day PO	EO	0	3	0.3	3
	CG	0	3	0.9	2.4
	Sham	0	3	1.3	2.4

## Discussion

In this study, the effects of essential oil from leaves of *L. gracilis* Schauer during the healing of rat skin lesions were investigated, whereby inflammation, angiogenesis, extracellular matrix formation, and wound contraction were evaluated. The proposed experimental model has been used to study the biological mechanisms involved in the healing of skin wounds^2^^,^ 6 ^,^ 22 ^-^ 25. An ointment containing 10% of essential oil was prepared, used topically on wounds for 21 consecutive days and tested for healing potential. That concentration was chosen based on pilot tests where anti-inflammatory effects and early skin wound contraction in rats were observed. Furthermore, a previous study showed that ointment containing 10% *Ximenia americana* showed anti-inflammatory effects and promotes early skin wound contraction in rats^6^.

The chemical composition of *L. gracilis* oil shows quantitative fluctuations of its major components, which depend on genetic and environmental conditions where the plant is cultivated^26^. The essential oil constituents are very unstable at light intensity and temperature. Consequently, the year season and the time of harvesting may directly or indirectly influence the secondary metabolism processes, which results in its quantitative and qualitative variations^27^. The chemical constituents analysis of the tested essential oil identified thymol and carvacrol as major substances present in the essential oil of *L. gracilis* (Fig. 1). In addition to those compounds, Bitu *et al*. also identified p-cymene, γ-terpinene and 4-methoxy-acetophenone as major components at different locations in the *Caatinga Pernambucana* 28.

Previous studies have reported therapeutic potential of *L. gracilis* constituents. The antimicrobial and antifungal activities of the essential oil from *L. gracilis* leaves were confirmed by experiments carried out by Pessoa et al. using bacteria (*Staphylococcus aureus* and *Escherichia coli)* and fungi (*Aspergillus niger* and *Penicillium* sp.)^29^. Thymol and carvacrol have been shown anti-inflammatory properties *in vitro* and *in vivo* in several cells and animal models^30^. Riella *et al.* demonstrated healing activity of thymol when added to a collagen-based film in skin wound rats^31^. Carvacrol presented inhibitory effects on leukocyte migration in experimental models of ear edema and carrageenan-induced pleurisy, as well as in *in vitro* chemotaxis^32^.

With the purpose of smoothing or softening the skin and even making it more flexible, emollient products such as petroleum jelly and anhydrous lanolin have been added to the composition of the ointment of *Lippia* sp.^33^. Lanolin has a moisturizing, protective, dispersing, adherent, and plasticizing action^34^. Other manipulations have been found in the literature. For instance, a *L. gracilis* essential oil at a concentration of 0.05% incorporated into dimethyl sulfoxide (DMSO) was topically applied on rat skin wounds. The healing time of the wounds was similar to the positive CG treated with dexamethasone^35^.

Healing of tissue injuries corresponds to the restoration of normal anatomical continuity in areas with tissue damage and involves processes of regeneration and repair. During skin wound healing, it is possible to observe the restoration of structure and function (regeneration) in the epidermis, as well as the replacement of the injured tissue by another that structurally and functionally differs from the original tissue (repair) in the dermis. It involves a complex combination of biochemical and cellular events that lead to the structural and functional reconstruction of compromised tissue^35^.

In the first response to injury (inflammatory phase), hemostasis takes place with its characteristics of vasoconstriction mediated by vasoactive factors. It is followed by vasodilation, accompanied by extravasation of cells, fibrinogen and coagulation elements, along with platelet thrombus formation, activation of the coagulation cascade and plug formation of fibrin. Those events give rise to a temporary extracellular matrix that facilitates the entry of defense cells, endothelial cells and fibroblasts into the wound^5^^,^ 36. Inflammation with leukocyte migration then occurs within a few hours, with neutrophils being the first to reach the injured area, followed by macrophages. Several inflammatory mediators are released, which is orchestrated on a large scale by platelets (PDGF, TGF-β), neutrophils (IL-1α, IL-1β, IL-6, IL-8, TNF-α), and macrophages (IL-1α, IL-1β, IL-6, TNF-α). Macroscopically, in the first days, the presence of exudate and crust formation, a combination of wound fluid, degraded neutrophils, and denatured tissue are observed in the region^5^^,^ 36.

In this study, a light to moderate crust formation not filling the entire wound area was observed in wounds of EO group, whereas a more intense and complete crust formation was seen in the CG and Sham groups. The inflammatory infiltrate formed by mononuclear and polymorphonuclear cells was moderate in EO group, while it was intense in the CG and Sham groups. At the 7th postoperative day, these crusts were mild in EO group and moderate to intense in CG and Sham groups. Moreover, a more intense inflammation was seen in the CG and Sham groups (Table 1). These data point to an anti-inflammatory action of the essential oil from *L. gracilis* leaves, as observed by both less inflammatory response and crust formation in EO group. Studies on the anti-inflammatory activity of that oil were developed by Mendes *et al*.^18^ in an experimental model of paw edema in rats. It was demonstrated that oral administration of EO from *L. gracilis* leaves at doses of 50, 100, and 200 mg·kg^–1^, exhibits anti-inflammatory activity in rat paw edema and analgesic effect in abdominal contractions in mice. Accordingly, scar formation is closely related to the chronicity of the inflammatory infiltration during wound healing, and this effect may be associated with the ability of these compounds to modulate the initial inflammatory phase^20^.

The proliferative phase (second scarring phase) involves several events, including angiogenesis, fibroplasia, contraction, and epithelialization^36^. Neoangiogenesis is the process of formation of new blood vessels. It starts around the 3rd day after the injury, being responsible for tissue nutrition, as well as for the increased supply of cells such as macrophages and fibroblasts to the wound site, characterizing secondary intention healing and granulation tissue^37^. The combination of new capillaries, fibroblasts and collagen forms the macroscopically bright red granulation tissue. It has been shown that the monoterpenes carvacrol and thymol stimulated reepithelialization, angiogenesis, and formation of granulation tissue and collagen fiber deposition in various histological analyses. Furthermore, carvacrol was able to induce angiogenesis by increasing the expression of vascular endothelial growth factor (VEGF), whereas both monoterpenes also increased TGF-β *in vitro* 38 ^,^ 39.

In this experiment, the group treated with essential oil from *L. gracilis* leaves showed a significant increase in angiogenesis on days 4 and 7 after surgery when compared to the other groups (Fig. 3). Meanwhile, an increase in collagen fibers density at the 7th and 14th postoperative days, as well as an improvement in collagen fibers orientation at the 14th and 21th days were noticed in skin wounds of the EO group (Fig. 4). Moreover, the essential oil promoted an earlier wound contraction when compared to CG and Sham groups from the 7th postoperative day. Similar results were found in studies with other terpenes, in which a higher number of blood vessels at the 7th day after surgery and improved collagen deposition were reported after treatment with a 10% ointment of aroeira oil (*Schinus terenbenthifolia* Radii)^5^.

The third phase of healing is the most clinically relevant and involves remodeling/maturation. The transition from ECM to scar requires remodeling with a decrease in type III collagen content. The predominant type III collagen in the fresh wound is replaced by type I collagen produced by fibroblasts. Tissue remodeling is a balance between the expression of proteolytic enzymes such as metalloproteinases and tissue inhibitors of metalloproteinases (TIMPs), on which growth factors contained in the ECM play a key role. Growth factors involved in that modulation include TGF-β, PDGF and IL-1. In addition to ECM remodeling, TIMPs also act in the healing process by inhibiting angiogenesis and inducing apoptosis^40^.

It was reported that thymol is able to promote the complete replacement of type III collagen for type I collagen in 14 days, indicating a normal and dynamic collagenization^31^. In this study, from the 7th day after operation, the collagen fibers were more organized, thickened and elongated in the EO group when compared to CG and Sham groups (Fig. 4). Furthermore, At the 21th postoperative day, both EO and CG groups presented complete granulation tissue formation with no inflammatory process and lower cellularity, while the Sham group still presented a mild to moderate inflammatory process (Table 1). Thus, this study supports the idea that the essential oil from *L. gracilis* leaves could improve skin wound healing.

In this study, macroscopic, histomorphological, and histomorphometric parameters were used to characterize the skin wound healing. As wound healing is a complex process whereby several cells and molecules including growth factors, enzymes, cytokines, and chemokines interact in a spatiotemporal coordinated manner^35^, a limitation of this study was that it did not evaluate some of these factors. In order to better understand the cellular and molecular mechanisms by which essential oil from of *L. gracilis* leaves act to improve skin wound healing, more methods such as immunohistochemistry, western blotting and gene expression analysis, would be essential to these purposes. Nevertheless, this study paves the way for future studies that could use these methods and other would healing markers.

## Conclusion

The treatment of skin lesions with essential oil from the leaves of *L. gracilis* Schauer in 10% ointment accelerates the initial stages of healing, reduces inflammation, and increases angiogenesis, collagen fibers density, and wound contraction in rats.

## References

[1] Mendonça FAS, Passarini JR, Esquisatto MAM, Mendonça JS, Franchini CC, Santos GMT (2009). Effects of the application of *Aloe vera* (L.) and
microcurrent on the healing of wounds surgically induced in Wistar
rats. Acta Cir Bras.

[2] Leite AGB, Estevão LRM, Silva CJFL, Lima JLS, Bulhões AAVC, Soares EBA, Evêncio-Neto J (2020). Avaliação morfo-histológica e morfo-histométrica de feridas
cutâneas tratadas com *Sphagneticola trilobata* (L.) Pruski
em ratos. Arq Bras Med Vet Zootec.

[3] Estevão LRM, Mendonça FS, Baratella-Evêncio L, Simões RS, Barros MEG, Arantes RME, Rachid MA, Evêncio-Neto J (2013). Effects of aroeira (*Schinus terebinthifolius*
Raddi) oil on cutaneous wound healing in rats. Acta Cir Bras.

[4] Estevão LRM, Medeiros JP, Simões RS, Arantes RME, Rachid MA, Silva RMG, Mendonça FS, Evêncio-Neto J (2015). Mast cell concentration and skin wound contraction in rats
treated with Brazilian pepper essential oil (*Schinus
terebinthifolius Raddi*). Acta Cir Bras.

[5] Estevão LRM, Simões RS, Cassini-Vieira IP, Canesso MCC, Barcelos LS, Rachid MA, Câmara CAG, Evêncio-Neto J (2017). *Schinus terebinthifolius* Raddi (Aroeira) leaves oil
attenuates inflammatory responses in cutaneous wound healing in
mice. Acta Cir Bras.

[6] Souza JC, Estevão LRM, Baratella-Evêncio L, Vieira MGF, Simões RS, Florencio-Silva R, Evêncio-Luz L, Evêncio-Neto J (2017). Mast cell concentration and skin wound contraction in rats
treated with *Ximenia americana* L. Acta Cir Bras.

[7] Piriz MA, Lima CAB, Jardim VMR, Mesquita MK, Souza ADZ, Heck RM (2014). Plantas medicinais no processo de cicatrização de feridas: uma
revisão de literatura. Rev Bras Plantas Med.

[8] Oliveira DR, Leitão GG, Santos SS, Bizzo HR, Lopes D, Alviano CS, Alviano DS, Leitão SG (2006). Ethnopharmacological study of two *Lippia* species
from Oriximiná, Brazil. J Ethnopharmacol.

[9] Pascual ME, Slowing K, Carretero E, Mata DS, Villar A (2001). Lippia: traditional uses, chemistry and pharmacology: A
review. J Ethnopharmacol.

[10] Albuquerque UP, Medeiros PM, Almeida ALS, Monteiro JM, Lins EMF, Melo JG, Santos JP (2007). Medicinal plants of the *caatinga* (semi-arid)
vegetation of NE Brazil: a quantitative approach. J Ethnopharmacol.

[11] Santos MM, Peixoto AR, Pessoa ES, Nepa HBS, Paz CD, Souza AVV (2014). Estudos dos constituintes químicos e atividade antibacteriana do
óleo essencial de *Lippia gracilis* a *Xanthomonas
campestris* pv. viticola “*in vitro”*. Summa Phytopathol.

[12] Guimarães AG, Gomes SVF, Moraes VRS, Nogueira PCL, Ferreira AG, Blank AF, Santos ADC, Viana MD, Silva GH, Quintans LJ (2012). Phytochemical characterization and antinociceptive effect of
*Lippia gracilis* Schauer. J Nat Med.

[13] Fernandes LCB, Albuquerque CC, Sales R, Oliveira FFM, Gurgel EP, Mesquita MV, Silva MDS (2015). Fungitoxicidade dos extratos vegetais e do óleo essencial de
*Lippia gracilis* Schauer sobre o fungo
*Monosporascus cannonballus* Pollack e
Uecker. Summa Phytopathol.

[14] Oliveira TNS, Silva-Filho CMS, Malveira EA, Aguiar TKB, Santos HS, Albuquerque CC, Morais MB, Teixeira EH, Vasconcelos MA (2021). Antifungal and antibiofilm activities of the essential oil of
leaves from *Lippia gracilis* Schauer against phytopathogenic
fungi. J Appl Microbiol.

[15] Ugulino ALN, Mendonça-Júnior AF, Rodrigues APMS, Santos AB, França KRS, Cardoso TAL, Prado-Júnior LS (2018). Inhibition effect of vegetable oils on the mycelial growth of
*Macrophomina phaseolina* (Tassi.). Goid J Agric Sci.

[16] Ferraz RPC, Bomfim DS, Carvalho NC, Soares MBP, Silva TB, Machado WJ, Prata APN, Costa EV, Moraes VRS, Nogueira PCL, Bezerra DP (2013). Cytotoxic effect of leaf essential oil of *Lippia
gracilis* Schauer (Verbenaceae). Phytomedicine.

[17] Guilhon CC, Raymundo LJRP, Alviano DS, Blank AF, Arrigoni-Blank MF, Matheus ME, Cavalcanti SCH, Alviano CS, Fernandes PD (2011). Characterization of the anti-inflammatory and antinociceptive
activities and the mechanism of the action of *Lippia
gracilis* essential oil. J Ethnopharmacol.

[18] Mendes SS, Bomfim RR, Jesus HCR, Alves PB, Blank AF, Estevam CS, Antoniolli AR, Thomazzi SM (2010). Evaluation of the analgesic and anti-inflammatory effects of the
essential oil of *Lippia gracilis* leaves. J Ethnopharmacol.

[19] National Council for the Control of Animal Experimentation
(CONCEA) National Council for the Control of Animal Experimentation.

[20] van Den Doll H, Kratz PD (1963). A generalization of the retention index system including linear
temperature programmed gas-liquid partition chromatography. J Chromatogr A.

[21] Leitão SG, de Oliveira DR, Sülsen V, Martino V, Barbosa YG, Bizzo HR, Lopes D, LF Viccini, Salimena FRG, Peixoto PHP, Leitão GG (2008). Analysis of the chemical composition of the essential oils
extracted from *Lippia lacunosa* Mart. & Schauer and
*Lippia rotundifolia* Cham. (Verbenaceae) by gas
chromatography and gas chromatography-mass spectrometry. J Braz Chem Soc.

[22] Estevão LRM, Cassini-Vieira P, Leite AGB, Bulhões AAVC, Barcelos LS, Evêncio-Neto J (2019). Morphological evaluation of wound healing events in the
excisional wound healing model in rats. Bio-protocol.

[23] Pessoa WS, Estevão LRM, Simões RS, Barros MEG, Mendonça FS, Baratella-Evêncio L, Evêncio-Neto J (2012). Effects of angico extract (*Anadenanthera
colubrina* var. *cebil*) in cutaneous wound
healing in rats. Acta Cir Bras.

[24] Pessoa WS, Estevão LRM, Simões RS, Barros MEG, Mendonça FS, Evêncio-Luz L, Baratella-Evêncio L, Florencio-Silva R, Sá FB, Evêncio-Neto J (2015). Fibrogenesis and epithelial coating of skin wounds in rats
treated with angico extract (*Anadenanthera colubrina* var.
*cebil*). Acta Cir Bras.

[25] Souza JC, Estevão LRM, Ferraz AA, Simões RS, Vieira MGF, Evêncio-Neto J (2019). Ointment of *Ximenes americana* promotes
acceleration of wound healing in rats. Acta Cir Bras.

[26] Gomes SVF, Nogueira PCL, Moraes VRS (2011). Aspectos químicos e biológicos do gênero *Lippia*
enfatizando *Lippia gracilis* Schauer. Eclet Quim J.

[27] Turek C, Stintzing FC (2013). Stability of essential oils: A review. Compr Rev Food Sci Food Saf.

[28] Bitu VCN, Fecundo HDTF, Costa JGM, Coutinho HDM, Rodrigues FFGR, Santana NM, Botelho MA, Menezes IRA (2014). Chemical composition of the essential oil of *Lippia
gracilis* Schauer leaves and its potential as modulator of
bacterial resistance. Nat Prod Res.

[29] Pessoa ODL, Carvalho CBM, Silvestre JOVL, Lima MCL, Neto RM, Matos FJA, Lemos TLG (2005). Antibacterial activity of the essential oil from *Lippia
aff. gracilis*. Fitoterapia.

[30] Salehi B, Mishra AP, Shukla I, Sharifi-Rad M, Contreras MDM, Segura-Carretero A, Fathi H, Nasrabadi NN, Kobarfard F, Sharifi-Rad J (2018). Thymol, thyme, and other plant sources: Health and potential
uses. Phytother Res.

[31] Riella KR, Marinho RR, Santos JS, Pereira-Filho RN, Cardoso JC, Albuquerque-Junior RLC, Thomazzi SM (2012). Anti-inflammatory and cicatrizing activities of thymol, a
monoterpene of the essential oil from *Lippia gracilis*, in
rodents. J Ethnopharmacol.

[32] Fachini-Queiroz FC, Kummer R, Estevao-Silva CF, Carvalho MDDB, Cunha JM, Grespan R, Cuman RKN (2012). Effects of thymol and carvacrol, constituents of *Thymus
vulgaris* L. essential oil, on the inflammatory
response. Evid Based Complement Alternat Med.

[33] Oliveira ML, Bezerra BM, Leite LO, Girão VC, Nunes-Pinheiro DC (2014). Topical continuous use of *Lippia sidoides* Cham.
essential oil induces cutaneous inflammatory response, but does not delay
wound healing process. J Ethnopharmacol.

[34] Coca KP, Abrão ACFV (2008). An evaluation of the effect ov lanolin in healing nipple
injuries. Acta Paul Enferm.

[35] Gonzalez ACO, Costa TF, Andrade ZA, Medrado ARAP (2016). Wound healing - A literature review. An Bras Dermatol.

[36] Krafts KP (2010). Tissue repair: The hidden drama. Organogenesis.

[37] Demidova-Rice TN, Durham JT, Herman IM (2012). Wound healing angiogenesis: Innovations and challenges in acute
and chronic wound healing. Adv Wound Care.

[38] Matluobi D, Araghi A, Maragheh BFA, Rezabakhsh A, Soltani S, Khaksar M, Siavashi V, Feyzi A, Bagheri HS, Rahbarghazi R, Montazersaheb S (2018). Carvacrol promotes angiogenic paracrine potential and endothelial
differentiation of human mesenchymal stem cells at low
concentrations. Microvasc Res.

[39] Costa MF, Durço AO, Rabelo TK, Barreto RSS, Guimarães AG (2019). Effects of carvacrol, thymol and essential oils containing such
monoterpenes on wound healing: A systematic review. J Pharm Pharmacol.

[40] Arpino V, Brock M, Gill SE (2015). The role of TIMPs in regulation of extracellular matrix
proteolysis. Matrix Biol.

